# Adrenal venous sampling in patients with ACTH-independent hypercortisolism

**DOI:** 10.1007/s12020-019-02038-0

**Published:** 2019-08-22

**Authors:** Eleni Papakokkinou, Hugo Jakobsson, Augustinas Sakinis, Andreas Muth, Bo Wängberg, Olof Ehn, Gudmundur Johannsson, Oskar Ragnarsson

**Affiliations:** 1 Department of Internal Medicine and Clinical Nutrition, Institute of Medicine at Sahlgrenska Academy, University of Gothenburg, Gothenburg, Sweden; 2 Department of Endocrinology, Sahlgrenska University Hospital, Gothenburg, Sweden; 3 Department of Radiology, Sahlgrenska University Hospital, Gothenburg, Sweden; 4 Department of Surgery, Institute of Clinical Sciences, Sahlgrenska Academy, University of Gothenburg, Sahlgrenska University Hospital, Gothenburg, Sweden

**Keywords:** Cushing’s syndrome, Adrenal venous sampling, Hypercortisolism, ACTH-independent, Autonomous cortisol secretion

## Abstract

**Purpose:**

To study the usefulness of adrenal venous sampling (AVS) in distinguishing unilateral from bilateral cortisol production in patients with ACTH-independent hypercortisolism and bilateral adrenal lesions, or morphologically normal adrenal glands.

**Methods:**

A retrospective analysis of ten consecutive patients with ACTH-independent hypercortisolism who underwent AVS at our institution between 2009 and 2017. Unilateral dominant cortisol production was defined as a side-to-side cortisol/aldosterone lateralization ratio >2.

**Results:**

Four of ten patients had overt Cushing’s syndrome. Of these, two had bilateral adrenal lesions on computed tomography and two had normal adrenal glands. One of the two patients with bilateral adrenal lesions had, based on the AVS, a unilateral dominant cortisol production. Following unilateral adrenalectomy the patient developed adrenal insufficiency. The other three patients were considered to have bilateral cortisol production and underwent bilateral adrenalectomy. Six patients had a mild autonomous cortisol secretion and bilateral adrenal lesions. Based on AVS, one patient was considered to have unilateral dominant cortisol production, underwent unilateral adrenalectomy and developed transient adrenal insufficiency postoperatively.

**Conclusions:**

AVS may contribute to appropriate treatment in patients with ACTH-independent hypercortisolism and bilateral adrenal lesions. In our series, AVS was helpful in the decision-making of two out of ten patients, avoiding chronic treatment with steroidogenesis inhibitors, or inappropriate bilateral adrenalectomy.

## Introduction

Adrenal Cushing’s syndrome (CS) is characterized by autonomous cortisol production with low plasma ACTH concentrations and is therefore defined as ACTH-independent CS. Overall, ACTH-independent CS accounts for 15–20% of endogenous CS where the most common cause is a unilateral cortisol producing adrenal adenoma [1]. Other rarer causes of ACTH-independent CS are primary bilateral macronodular adrenal hyperplasia (PBMAH) and primary pigmented nodular adrenocortical disease (PPNAD) (<2%) [2]. Approximately 10% of patients with ACTH-independent CS have bilateral adrenal lesions [3].

Mild autonomous cortisol secretion (MACS) is defined as a mild hypersecretion of cortisol in patients with adrenal incidentaloma but without the typical signs and symptoms of clinically overt hypercortisolism. MACS is present in 5–30% of patients with incidentally discovered adrenal adenomas [4]. Importantly, 9–17% of patients with adrenal incidentalomas have bilateral lesions [5, 6], and seem to have an even higher prevalence of MACS than patients with unilateral lesions [7].

While diagnostic work-up and treatment of patients with ACTH-independent CS and unilateral adenomas is usually straightforward, management of patients with bilateral adrenal lesions is more challenging since distinction between a functioning and nonfunctioning adrenal lesion cannot be reliably determined by specific radiological or clinical features [8]. The same applies to patients with ACTH-independent CS and morphologically normal adrenal glands.

Adrenal venous sampling (AVS) is an established method to distinguish unilateral from bilateral aldosterone overproduction [9]. The usefulness of AVS for differentiating unilateral from bilateral cortisol overproduction in patients with ACTH-independent hypercortisolism has however only been evaluated in two studies. Young et al. found AVS to be helpful for deciding treatment strategy in ten patients with ACTH-independent CS and bilateral adrenal masses [10] and, recently, Ueland et al. found that four of fourteen patients with MACS and bilateral adrenal lesions had unilateral cortisol hypersecretion [8]. Otherwise, the diagnostic value of AVS in this context is limited to case reports [11–19].

Hereby we report our experience using AVS in ten patients with ACTH-independent hypercortisolism.

## Material and methods

### Patients

This was a retrospective analysis of ten consecutive patients with ACTH-independent hypercortisolism that were subjected to AVS at our institution between 2009 and 2017. Eight patients had been referred for evaluation of incidentally found bilateral adrenal lesions and two due to clinically overt CS. All patients were initially evaluated clinically and biochemically (Table 1). In all patients, ACTH was measured at 8 a.m. and serum cortisol at midnight, an overnight 1 mg dexamethasone suppression test was performed, and 24 h urine was sampled for measurement of urinary free cortisol (UFC). All patients were evaluated with low-dose dexamethasone suppression test (LDDST; 0.5 mg every sixth hour for 2 days). MACS was defined as absence of typical clinical signs and symptoms of CS, pathological dexamethasone suppression test (>50 nmol/L) and normal UFC in patients with adrenal incidentaloma. In addition, at least two of the following needed to be fulfilled: (a) low or low normal ACTH concentrations in plasma (<4.0 pmol/L), (b) serum cortisol at midnight >100 nmol/L, and (c) serum cortisol >50 nmol/L following LDDST.

**Table 1:** Clinical, radiological and biochemical characteristics of the study population

Pat no	Age (year)	Sex	BMI (kg/m^2^)	Clinical presentation	Hypercortisolism	Main clinical features	Radiological findings	S- cortisol (nmol/L) after ONDST	ACTH (pmol/L)	UFC (times the ULN)	S-cortisol (nmol/L) at midnight	S-cortisol (nmol/L) after LDDST
**1**	55	F	26.0	Bilateral adrenal incidentaloma	MACS	Muscle weakness Hypertension	Bilateral adenomas: Right 32 × 18 mm	840	1.7	0.8	340	630
							Left 14 × 8 mm					
**2**	60	F	23.9	Bilateral adrenal incidentaloma	MACS	Hypertension Type 2 DM Muscle weakness	Bilateral adenomas: Right 28 × 22 mm	110	1.9	1.0	93	100
							Left 15 × 10 mm					
**3**	54	M	22.4	Bilateral adrenal incidentaloma	MACS	Hypertension Weight gain Decreased libido Impaired memory	Bilateral adenomas: Right 19 × 14 mm	190	3.2	0.8	140	120
							Left 28 × 25 mm					
**4**	61	F	33.0	Bilateral adrenal incidentaloma	MACS	Hypertension Obesity	Multiple adenomas, two in right adrenal and three in left, largest adenomas: Right 32 × 22 mm	563	<0.2	0.6	250	420
							Left 19 × 18 mm					
**5**	62	M	24.1	Bilateral adrenal incidentaloma	MACS	Hypertension Muscle weakness Impaired memory	Bilateral adenomas: Right 23 × 16 mm	120	3,6	0.9	120	120
							Left 34 × 36 mm					
**6**	60	F	30.5	Bilateral adrenal incidentaloma	MACS	Mild Cushingoid features Hypertension Type 2 DM	Bilateral adenomas: Right 23 × 15 mm	150	2.6	0.8	170	120
							Left 10 × 10 mm					
**7**	59	F	34.6	Bilateral adrenal incidentaloma	Moderate CS	Cushingoid features Hypertension Type 2 DM Fatigue Depression	Bilateral adenomas: Right 22 × 15 mm	610	1.1	1.3	420	560
							Left 64 × 50 mm					
**8**	57	M	34.9	Bilateral adrenal incidentaloma	Moderate CS	Cushingoid features Hypertension Osteoporosis Fatigue Depression	Bilaterally enlarged adrenal glands: Right 50 × 47 mm	550	0.9	4.7	570	250
							Left 41 × 32 mm					
**9**	39	F	23.6	CS	Moderate CS	Cushingoid features Depression Amenorrhea Osteoporosis Fatigue	Normal adrenal glands	677	1.3	1.6	560	747
**10**	11	F	40.8	CS	Severe CS	Cushingoid features Weight gain Decreased growth rate Hypertension	Normal adrenal glands	830	1.7	3.9	680	340
**11**	51	F	30.1	CS	Severe CS	Cushingoid features Weight gain Hypertension Type 2 DM Fatigue Muscle weakness	Mildly enlarged adrenal glands bilaterally with suspected 9 mm adenoma in the right adrenal gland	730	2.4/4.0/5.7	10.2	900	–

*F* female, *M* male, *AVS* adrenal venous sampling, *MACS* mild autonomous cortisol secretion, *BMI* body mass index, *CS* Cushing’s syndrome, *ACTH* adrenocorticotropic hormone at 8 a.m. (ref. 2.0–11 pmol/L), *UFC* 24-h urinary free cortisol, *ULN* upper limit of the normal range, *ONDST* 1 mg overnight dexamethasone suppression test, *LDDST* low-dose dexamethasone suppression test, *DM* diabetes mellitus type 2

In addition, one patient with suspected ACTH-independent Cushing’s syndrome, due to low to normal ACTH levels and a suspected 9 mm right-sided adrenal adenoma, underwent AVS during the study period. Eventually, this patient was found to have cyclic Cushing’s disease and was successfully treated with transsphenoidal pituitary adenomectomy. For illustrative purposes, the data for this patient is presented in Tables 1–3 but is otherwise excluded from the analyses.

**Table 2:** Results from adrenal venous sampling in 11 patients with hypercortisolism

	Peripheral	Vena cava	Left adrenal	Right adrenal	L SI	R SI	R/L gradient	L/R gradient
Patient 1								
Cortisol (nmol/L)	480	480	2400	6040				
Aldosterone (pmol/L)	54	79	564	539	10.4	10.0	2.6	
Noradrenaline (nmol/L)	–	0.8	22	16			3.6	
Adrenaline (nmol/L)	–	0.2	97	58			4.2	
DHEA-s (μmol/L)	1.7	2.2	2.2	3.8			1.5	
Patient 2								
Cortisol (nmol/L)	140	140	520	940				
Aldosterone (pmol/L)	52	60	228	307	4.4	5.9	1.3	
Noradrenaline (nmol/L)	–	–	–	–			–	
Adrenaline (nmol/L)	–	–	–	–			–	
DHEA-s (μmol/L)	0.76	0.69	0.9	1.0			1.6	
Patient 3								
Cortisol (nmol/L)	210	220	1710	1520				
Aldosterone (pmol/L)	78	90	1830	2260	23.5	29.0		1.4
Noradrenaline (nmol/L)	1.3	1.0	1.7	15				10
Adrenaline (nmol/L)	0.1	0.1	4.2	57				14
DHEA-s (μmol/L)	0.6	0.6	0.6	0.8				1.3
Patient 4								
Cortisol (nmol/L)	330	300	1650	2670				
Aldosterone (pmol/L)	141	208	559	1790	4.0	12.7		2.0
Noradrenaline (nmol/L)	–	–	–	–			–	
Adrenaline (nmol/L)	–	–	–	–			–	
DHEA-s (μmol/L)	–	–	–	–			–	
Patient 5								
Cortisol (nmol/L)	120	–	1320	1830				
Aldosterone (pmol/L)	<30	–	622	1960	20.7	65.3		2.3
Noradrenaline (nmol/L)	0.5	0.9	2.4	1.8			1.8	
Adrenaline (nmol/L)	0.3	0.1	7.0	159				16
DHEA-s (μmol/L)	1.6	1.6	1.9	1.6			1.6	
Patient 6								
Cortisol (nmol/L)	100	110	790	1450				
Aldosterone (pmol/L)	321	279	31500	5540	98.1	17.3	10.4	
Noradrenaline (nmol/L)	0.5	0.9	2.4	1.8			2.5	
Adrenaline (nmol/L)	<0.02	<0.02	6.8	6.3			2.0	
DHEA-s (μmol/L)	0.6	0.6	0.9	0.9			1.8	
Patient 7								
Cortisol (nmol/L)	540	570	1620	980				
Aldosterone (pmol/L)	57	96	318	1340	5.6	23.5		7.1
Noradrenaline (nmol/L)	1.9	2.2	1.1	8.4				12.5
Adrenaline (nmol/L)	0.3	<0.16	0.2	40				330
DHEA-s (μmol/L)	7	7	7.7	7.4				1.6
Patient 8								
Cortisol (nmol/L)	390	390	1000	1530				
Aldosterone (pmol/L)	46	50	268	375	5.8	8.1	1.1	
Noradrenaline (nmol/L)	1.5	1.6	3.8	2.2			2.6	
Adrenaline (nmol/L)	0.2	<0.16	18	13			2.1	
DHEA-s (μmol/L)	8.4	8.1	8.6	9.3			1.0	
Patient 9								
Cortisol (nmol/L)	510	490	8200	16,300				
Aldosterone (pmol/L)	<30	<30	585	1410	19.5	47.0		1.2
Noradrenaline (nmol/L)	0.6	0.4	5.7	4.4			2.6	
Adrenaline (nmol/L)	<0.02	<0.02	29	22			2.6	
DHEA-s (μmol/L)	3.1	2.9	4.7	6.1			1.5	
Patient 10								
Cortisol (nmol/L)	210	230	5030	3930				
Aldosterone (pmol/L)	1220	302	245	186	^*^	^*^	1.0	
Noradrenaline (nmol/L)	1.1	0.4	3.2	3.6				1.5
Adrenaline (nmol/L)	0.3	<0.16	26	55				2.7
DHEA-s (μmol/L)	0.13	0.12	0.2	0.2				1.3
Patient 11								
Cortisol (nmol/L)	280	260	6240	12300				
Aldosterone (pmol/L)	230	254	3870	16000	16.8	69.6		2.1
Noradrenaline (nmol/L)	0.3	<0.18	2.2	2.6			1.7	
Adrenaline (nmol/L)	<0.16	<0.16	12	13			1.8	
DHEA-s (μmol/L)	0.9	0.8	2.1	3.0			1.4	

*DHEA-s* dehydroepiandrosterone-sulfate, *L* left, *R* right, *SI* selectivity index

^*^Not calculated due to laboratory error of the peripheral sample

**Table 3:** Results from the adrenal venous sampling (AVS), treatment, histopathological findings and outcome

Patient no	Interpretation of the AVS	Treatment	Pathology	Follow-up months	Treatment response
1	Right-sided hypercortisolism	Right-sided laparoscopic adrenalectomy	Adrenal adenoma	31	Postoperative adrenal insufficiency, 13 kg weight reduction, improved blood pressure and significantly increased muscle strength
2	Bilateral hypercortisolism	Steroidogenesis inibitor	–	85	Significantly improved general well-being and insulin sensitivity
3	Bilateral hypercortisolism	Steroidogenesis inibitor for 4 months, thereafter surveilance	–	86	No improvement after 4 months on medical therapy. No progress of symptoms during active surveilance
4	Bilateral hypercortisolism	Steroidogenesis inibitor	–	12	Improved general well-being
5	Bilateral hypercortisolism	Steroidogenesis inibitor for 4 months, thereafter surveillance	–	23	No improvement on medical therapy, rather deterioration with lethargy and malaise
6	Bilateral hypercortisolism	Steroidogenesis inibitor for 6 months, thereafter surveillance	–	50	Improved general well-being on medical treatment, discontinued due to side-effects. During active surveillance, 25 kg weight reduction and resolusion of DM
7	Left-sided hypercortisolism	Left-sided adrenalectomy	Adrenal adenoma	51	Postoperative adrenal insufficiency. Resolution of all CS features, including hypertension, DM and depression
8	Bilateral hypercortisolism	Bilateral laparoscopic adrenalectomy	PBMAH	52	20 kg weight reduction. Improved blood pressure and general well-being
9	Bilateral hypercortisolism	Bilateral laparoscopic adrenalectomy	PPNAD	107	Resolution of all CS features. Restoration of regular menstruation and improved general well-being
10	Bilateral hypercortisolism	Bilateral laparoscopic adrenalectomy	PPNAD	6	Resolution of all CS features
11	Bilateral hypercortisolism	TSS	ACTH-positive pituitary adenoma	27	Postoperative adrenal insufficiency and resolution of CS features

Follow-up months from adrenal venous sampling until the last visit or until 31 December 2017

*CS* Cushing’s syndrome, *ACTH* adrenocorticotropic hormone, *CS* Cushing’s syndrome, *AVS* adrenal venous sampling, *DM* diabetes mellitus type 2, *PBMAH* primary bilateral macronodular hyperplasia, PPNAD primary pigmented nodular adrenocortical disease, TSS transsphenoidal surgery

### Adrenal venous sampling

AVS was performed on the second day after 48-h dexamethasone suppression (0.5 mg every sixth hour). The AVS procedure was performed as previously described [20]. In short, the right femoral vein was punctured and a SIM 1 catheter, Cobra, or Shepherd Hook was used to localize and draw blood from the right adrenal vein. Next, the left adrenal vein was localized and sampled by using a SIM 2 or SIM 3 catheter. Finally, blood samples from the inferior vena cava and a peripheral vein were drawn.

Concentrations of serum cortisol (*n* = 10), aldosterone (*n* = 10), DHEAS (*n* = 9), plasma adrenaline (*n* = 8), and noradrenaline (*n* = 8) were measured. The adequacy of the cannulation was based on the adrenal to peripheral vein ratio of aldosterone of >2, in accordance with the selectivity index used during AVS in patients with primary aldosteronism (without cosyntropin use) [9, 21]. The lateralization of the cortisol secretion was determined by calculation of the side-to-side adrenal vein cortisol concentration to aldosterone, adrenaline, noradrenaline, and DHEAS, respectively [(Cortisol/Reference hormone_Dominant adrenal vein_)/(Cortisol/Reference hormone_Non-dominant adrenal vein_)]. Since no consensus exists on how to optimally define laterality during AVS in patients with CS, each patient was evaluated individually. However, in general, a patient was considered to have a unilateral dominant cortisol production when the side-to-side lateralization ratio was >2, when using aldosterone as a reference hormone, especially in conjunction with a concordant side-to-side lateralization ratio >2 using the other three hormones as reference. When the concentration of the aldosterone, adrenaline, or noradrenaline was unmeasurably low, the lower limit of detection was used for calculation of selectivity and lateralization.

The decision for the treatment in all cases was based on the severity of hypercortisolism, laboratory data, imaging findings, as well as findings from the AVS and the patient’s preference.

### Assays

Serum cortisol was measured by using competitive electrochemiluminescence immunoassays; Roche Modular E (2009–2011; CV 5–7%), Roche Cobas, Cortisol 1 (2011–2015; CV 3–4%), and Roche Cobas, Cortisol-II (2015–2017; CV 2–3 %). Serum aldosterone was measured with radioimmunoassays; Siemens Coat-A-Count (November 2009–October 2014; CV 6–10%) and DiaSorin Liaison (November 2014–2017; CV 8–13%). Serum DHEAS was measured with a radioimmunoassay Perkin- Elmer Coat-A-Count, DPC Scandinavia AB (2009–2010; CV 12%), and with an immunoenzymatic method Beckman Coulter (2010–2017; CV 10%). Plasma ACTH was measured with an ELSA immunoradiometric assay (2009–2016; CV 6.8–14%) and with an electrochemiluminescence immunoassay for adrenocorticotropic hormone on a Cobas E Analyzer (2016–2017; CV 3.04–3.8%). Plasma adrenaline and noradrenaline were measured with high performance liquid chromatography with electrochemical detection (Chromeleon computer system; 2009–2017; CV for adrenaline 7–8% and 6% for noradrenaline).

### Statistical methods

The statistical analyses were performed with SPSS, version 22.0 for Windows. Continuous variables are presented as median (25–75 percentiles; range) and categorical variables as number (%).

## Results

### Baseline characteristics

Eleven patients, eight women and three men, were studied with AVS between 2009 and 2017. The median (25–75 percentiles; range) age at diagnosis was 57 (51–61; 11–62) years (Table 1).

Eight patients were initially referred for evaluation of adrenal incidentaloma, six of these had MACS and two had clinically overt CS (Table 1). Two patients were referred due to overt ACTH-independent CS. Thus, in total, four patients had clinically overt CS. Two of these had bilateral adrenal lesions and two had normal adrenal glands. All patients with MACS had bilateral adrenal lesions.

All patients were considered to be successfully catheterized. Nine of ten patients had adrenal to peripheral vein ratio of aldosterone >2 (selectivity index). The mean selectivity index was 31 (range 4.0–98) and 24 (range 5.9–65) for the left and right adrenal veins, respectively (Table 2). In addition, the AVS in one patient (subject 10), with selectivity index <2, was considered to be successful based on more than ten times higher concentrations of cortisol, and adrenaline, from the adrenal veins compared to peripheral vein.

### Patients with MACS

**Fig. 1:**
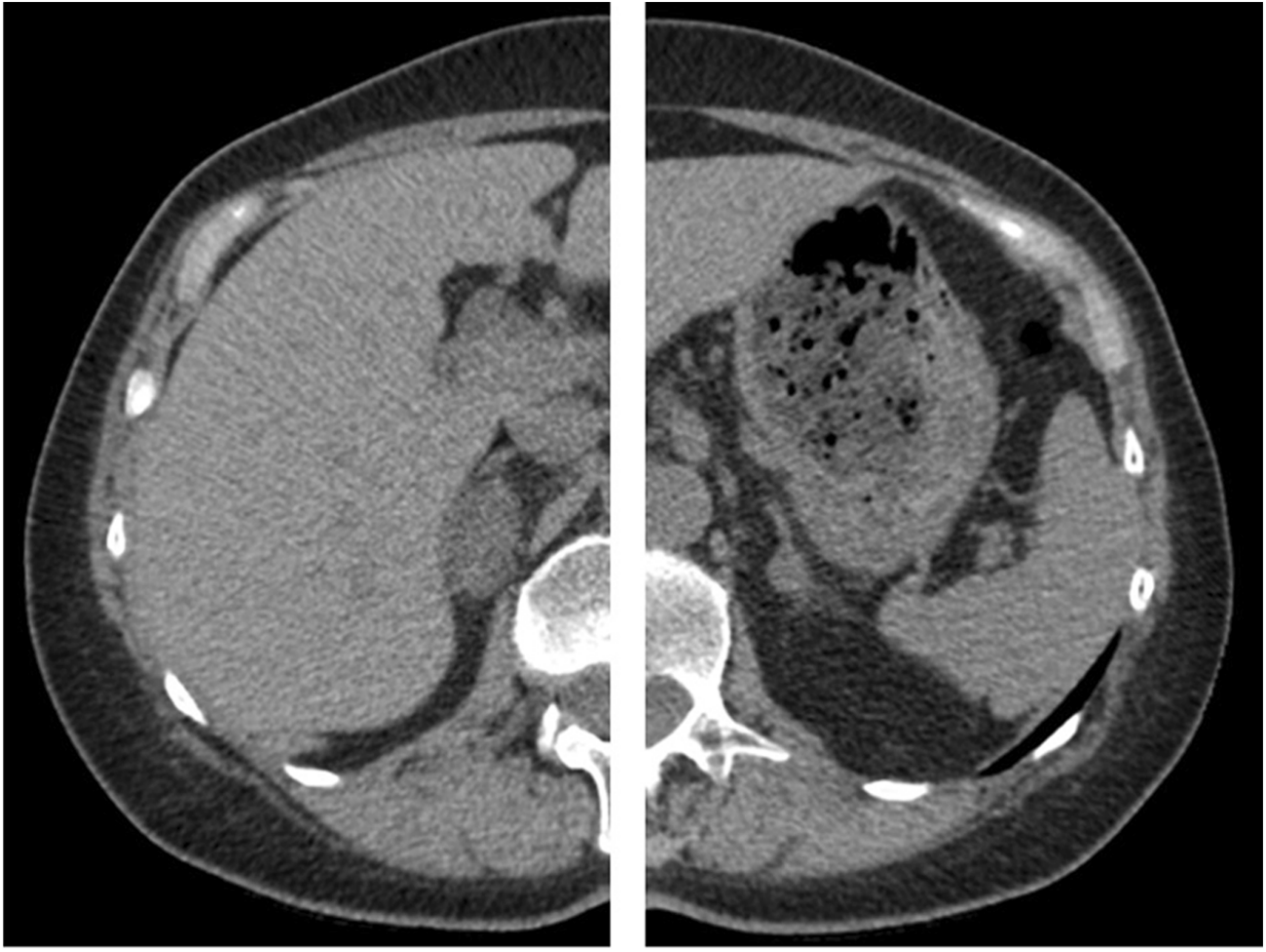
Patient with mild autonomous cortisol secretion (MACS) and bilateral adrenal lesions (right lesion 32 × 18 mm and left lesion 14 × 8 mm; Subject 1). AVS showed right-sided cortisol overproduction and right-sided adrenalectomy was performed. Postoperatively the patient developed transient adrenal insufficiency

Four women and two men had bilateral adrenal incidentalomas and MACS (Subjects 1–6; Tables 1–3). All had normal UFC and none had suppressed serum cortisol following overnight low-dose dexamethasone test. One patient (Subject 1; Fig. 1) was considered to have dominant cortisol production from the right adrenal gland based on a right to left cortisol/aldosterone gradient of 2.6 (Subject 1; Tables 1–3). Right to left cortisol/noradrenaline, cortisol/adrenaline and cortisol/DHEAS gradients were 3.6, 4.2, and 1.5, respectively (Table 2). The patient underwent unilateral adrenalectomy and developed adrenal insufficiency postoperatively. Histopathological examination showed a benign adrenal adenoma.

Based on the side-to-side gradients, four patients were considered to have bilateral MACS. In addition, one patient (Subject 6; Table 2) with concomitant primary aldosteronism was considered to have bilateral MACS based on relatively low side-to-side cortisol/noradrenaline, cortisol/adrenaline, and cortisol/DHEAS gradients. None of these five patients had overt CS and received therefore either medical treatment or active surveillance (Tables 2 and 3).

### Patients with overt hypercortisolism

**Fig. 2:**
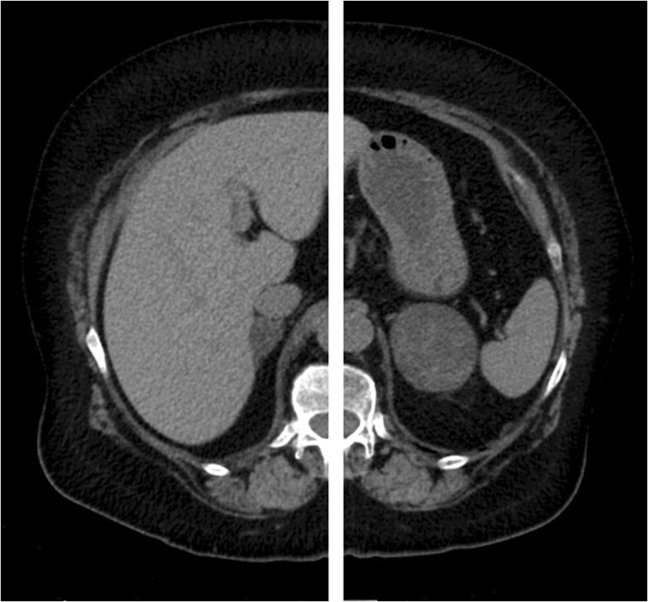
Patient with overt CS and bilateral adrenal lesions (right lesion 22 × 15 mm and left lesion 64 × 50 mm; Subject 7). AVS showed left-sided cortisol production and the patient underwent left-sided adrenalectomy. Postoperatively the patient developed adrenal deficiency. After 51 months of follow-up the patient has remained in remission

**Fig. 3:**
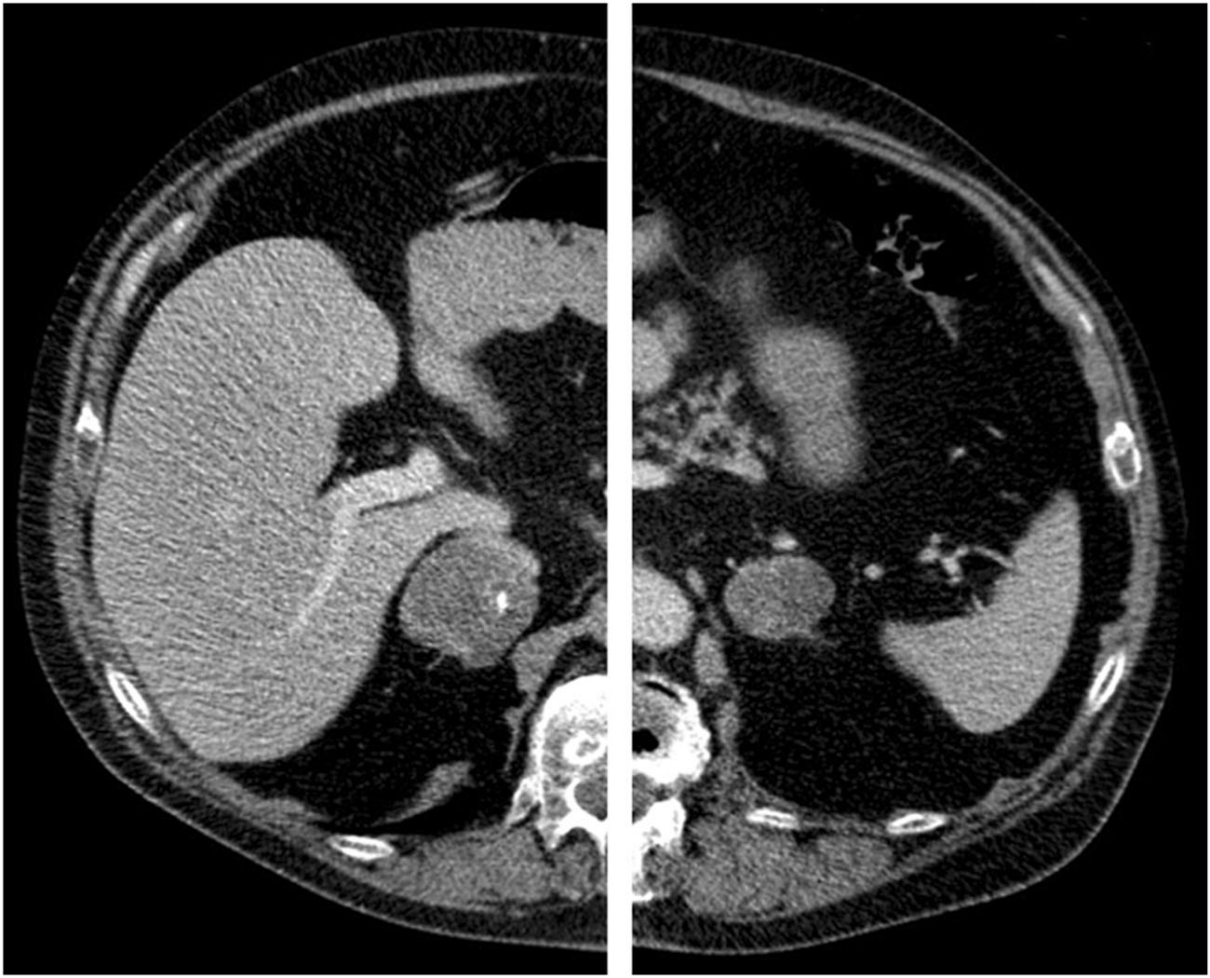
Patient with CS and bilateral lesions (right lesion 50 × 47 mm and left lesion 41 × 32 mm; Subject 8). AVS showed bilateral cortisol production, and bilateral adrenalectomy was performed. The histopathological diagnosis was PBMAH

Two patients had overt hypercortisolism and bilateral adrenal lesions (Subjects 7 and 8; Figs. 2 and 3). One of them had unilateral dominant cortisol production based on a left-to-right cortisol/aldosterone gradient of 7.1 and underwent left-sided adrenalectomy (Subject 7; Tables 1–3). The histopathological diagnosis was benign adrenal adenoma. Postoperatively, the patient developed adrenal insufficiency and all clinical features of hypercortisolism resolved. The other patient (Subject 8; Tables 1–3) had bilateral cortisol overproduction and was treated with bilateral adrenalectomy. The histopathological diagnosis was PBMAH.

Two patients (Subjects 9 and 10; Tables 1–3) had overt hypercortisolism and morphologically normal adrenal glands on CT. Both had bilateral cortisol overproduction and were treated with bilateral adrenalectomy. Histopathological examination confirmed PPNAD in both cases.

## Discussion

In our cohort, which included patients with ACTH-independent hypercortisolism and bilateral adrenal lesions or normal adrenal glands, AVS contributed to an appropriate choice of unilateral adrenalectomy in two out of ten patients. The study demonstrates that AVS in these cases was valuable, and facilitated avoidance of chronic treatment with steroidogenesis inhibitors or inappropriate bilateral adrenalectomy. Furthermore, two of our patients had normal adrenal glands on CT. In these cases, the AVS was useful for two reasons. Firstly, it confirmed that the excessive cortisol production was of adrenal origin, and secondly, it excluded laterality and thereby supporting the choice of bilateral adrenalectomy.

Unilateral minimally invasive adrenalectomy is the primary treatment of choice for patients with CS caused by unilateral cortisol producing adrenal adenomas. On the other hand, no consensus exists regarding treatment of patients with ACTH-independent CS and bilateral or undetectable lesions. Diagnosing PPNAD preoperatively can be difficult since other signs of the Carney complex are not always present, not all patients have the otherwise typical PRKAR1A mutation [22], and variations in the appearance of the adrenal glands on CT. In fact, more than half of patients with PPNAD have either morphologically normal adrenal glands, or micronodules that can be difficult to detect, on CT. Similarly, preoperative diagnosis of PBMAH is difficult and can easily be confused with bilateral adrenal adenoma, either functioning or non-functioning. Patients with either PPNAD or PBMAH are usually treated by bilateral adrenalectomy, although unilateral adrenalectomy has been reported to be efficacious in some of them [3, 23, 24]. The potential benefit of unilateral adrenalectomy in patients with PBMAH is a preserved adrenal function, while a risk for subsequent recurrence remains. In a retrospective study of 15 patients with PBMAH who underwent unilateral adrenalectomy, where the larger gland according to CT was removed, recurrence was observed in two of fifteen patients after a follow-up of 7 and 8 years respectively [25]. Our patient with PBMAH (Subject 8) who underwent bilateral adrenalectomy had a slightly larger right adrenal gland on a CT scan (50 × 47 mm versus 41 × 32 mm). In some centers, based on the size difference, our patient would have been treated with right-sided adrenalectomy. The AVS, however, with a side-to-side cortisol/aldosterone gradient of 1.1, demonstrated a bilateral cortisol production, supporting the choice of bilateral adrenalectomy.

In some centers, adrenocortical scintigraphy with ^131^I-methylnorcholesterol (NP-59) is used to evaluate if adrenal activity in patients with CS is uni- or bilateral [22, 25–27]. In primary aldosteronism, the sensitivity of this method depends on the size of the adenoma, and is not helpful in cases of small adenomas [28]. Furthermore, since the availability of this method is limited in many countries, AVS could instead be a valuable option to assess whether the cortisol production in these patients is uni- or bilateral. It should however be stressed that AVS is an invasive and technically difficult procedure that should only be performed by an experienced and dedicated interventionist [20].

In patients with primary aldosteronism, cortisol concentrations in the right and left adrenal veins as well as in a peripheral vein are used to evaluate whether the catheterization is successful. A widely accepted criteria for successful catheterization is a central to peripheral gradient ≥5 under ACTH stimulation, i.e. that cortisol concentrations in the adrenal veins should be at least five time higher than the concentration in a peripheral vein, and ≥2 without ACTH stimulation [9, 21]. How successfulness of the cannulation during AVS is best evaluated in patients with ACTH- independent CS is however not known. The AVS protocols from previous reports varies significantly [8, 10–13, 16–18]. In some cases dexamethasone suppression prior to AVS has been used in order to suppress physiologic endogenous ACTH secretion [8, 10, 16] while in others it has not [13]. AVS has also been performed during simultaneous ACTH stimulation [17]. The interpretation of adequate catheterization varies as well. In one report the radiographic localization of the catheter tip during collection of the adrenal samples was used [12]. In other reports measurements of noradrenaline and adrenaline [13], or only adrenaline [10, 16], were used for this purpose. In the report from the Mayo Clinic, a catheterization was defined as successful if the difference between plasma adrenaline concentration in an adrenal vein was higher than 100 pg/mL (0.56 nmol/L) compared to a peripheral vein [10]. In our cohort, adrenaline concentrations were measured in eight out of ten patients. If we had applied the same criteria, one cannulation would have been considered unsuccessful (Subject 7). The usefulness of catecholamines concentrations during AVS has, however, been questioned [29, 30] due to unacceptably wide side-to-side differences, as was observed in three out of nine of our patients (Subjects 3, 5, 7; Table 2), short half-life, as well as large inter-individual variations. Furthermore, catecholamines derive from the adrenal medulla and may therefore not, necessarily, represent the venous drainage from the adrenal cortex. However, in a recent study, plasma metanephrine in the adrenal veins and peripheral vein were used for the assessment of the catheterization. Metanephrine was suggested to be a promising reference hormone for this purpose, both due to its short half-life and that its secretion is not correlated to stress [8, 31].

Similarly, the criteria for evaluation of laterality in patients with hypercortisolism have not been established. In the report from the Mayo clinic, including 10 patients with ACTH-independent CS and bilateral adrenal masses [10], an adrenal vein to peripheral vein gradient greater than 6.5 was considered to be consistent with a cortisol-secreting adenoma and a cortisol lateralization ratio (side-to-side adrenal vein cortisol ratio) ≥2.3 consistent with unilateral dominant disease [10]. The same criteria for unilateral disease was used by Ueland et al. [8]. Applying the same criteria for lateralization in our cohort, one patient (Subject 7) would have been misclassified as having bilateral disease (left-to-right cortisol lateralization ratio 1.65). A possible explanation in that case was that the catheterization of the left adrenal vein was semi-selective, and illustrates simultaneously the importance of a collection of a reference hormone(s) in order to be able to take potential diluting effects into account. DHEAS was also collected during the AVS in our study. However, the use of DHEAS as a reference hormone is probably not suitable due to its long half-life (10 h).

To our knowledge, aldosterone has only been used once previously to correct for possible dilution [12]. Although we found side-to-side cortisol/aldosterone ratio to be useful in the two patients who eventually were treated by unilateral adrenalectomy, the role of measuring aldosterone remains to be further evaluated. The use of aldosterone as a reference hormone in order to correct the dilution differences may have limitations. Stress induced fluctuations in aldosterone secretion, that are minimized by ACTH stimulation during AVS for primary aldosteronism [9], cannot be eliminated during AVS in patients with ACTH-independent CS. Also, aldosterone has a shorter half-life (20 min) than cortisol (60–70 min) that may interfere in the interpretation of AVS findings. Furthermore, aldosterone and cortisol co-producing adenomas may be more common than previously thought [32], making interpretation of both successfulness as well as laterality difficult. In fact, one patient (Subject 6) in our cohort had a strikingly high aldosterone concentration from the left adrenal vein, due to a concomitant primary aldosteronism. Another patient had markedly elevated aldosterone concentrations from the peripheral vein (Subject 10), most probably explained by a laboratory error since repeated measurement of aldosterone and renin was normal.

A major limitation of this study is the small number of patients and the retrospective design. Although the same AVS protocol for the technical procedure was used throughout the study, the interpretation of the AVS results was not predefined, neither the criteria for adrenal vein selectivity nor laterality. Instead, each AVS was evaluated individually. In general, and without any firm scientific support, patients with side-to-side cortisol/aldosterone ratio >2 were considered to have unilateral dominant disease, especially when the side-to-side cortisol to adrenaline and noradrenaline, respectively, were also >2, with dominance on the same side. Both patients who fulfilled these criteria were operated with unilateral adrenalectomy and developed adrenal insufficiency postoperatively. However, it is important to emphasize that the beneficial effect of bilateral adrenalectomy in patients with overt CS, and apparent bilateral cortisol production according to the AVS, should be interpreted with caution due to lack of independent evidence of unilateral cortisol production with another modality such as adrenal scintigraphy. Another approach in such cases could be unilateral adrenalectomy, where the larger adrenal gland is removed, and the remaining gland would only be removed if the hypercortisolism remained. In fact, if this approach would have been used in subject 1, AVS would not have been necessary. Due to the limited size of the cohort, further studies are however needed before general recommendations concerning criteria for successful catheterization and significant laterality can be provided. Another limitation is that dexamethasone was not measured to assure adequate bioavailability and that more than one assay was used for most of the hormones during the study period. The latter does, however, unlikely affect the main results since these are based on quotients rather than concentrations.

In conclusion, this study on consecutive patients with ACTH-independent hypercortisolism showed that AVS contributed to appropriate unilateral adrenalectomy in two out of ten patients. Knowledge on the usefulness of AVS in patients with ACTH-independent hypercortisolism is still emerging, but the method may, in some cases, be a helpful tool for appropriate therapeutic decision, especially if other modalities are not available.

## Abbreviations

ACTH: adrenocorticotropic hormone
CS: Cushing’s syndrome
MACS: mild autonomous cortisol secretion
AVS: adrenal venous sampling
PBMAH: primary bilateral macronodular hyperplasia
PPNAD: primary pigmented nodular adrenocortical disease
LDDST: low-dose dexamethasone suppression test
DHEAS: dehydroepiandrosterone sulfate
BMI: body mass index
CT: computed tomography
